# Single-cell RNA sequencing reveals heterogeneous tumor and immune cell populations in early-stage lung adenocarcinomas harboring EGFR mutations

**DOI:** 10.1038/s41388-020-01528-0

**Published:** 2020-11-03

**Authors:** Di He, Di Wang, Ping Lu, Nan Yang, Zhigang Xue, Xianmin Zhu, Peng Zhang, Guoping Fan

**Affiliations:** 1 Shanghai Institute for Advanced Immunochemical Studies, ShanghaiTech University, Shanghai, 201210 China; 2 Shanghai Pulmonary Hospital, Department of Thoracic Surgery, School of Life Sciences and Technology, Tongji University, Shanghai, 200433 China; 3 Translational Center for Stem Cell Research, Tongji Hospital, Department of Regenerative Medicine, Tongji University School of Medicine, Shanghai, 200065 China; 4 PharmaLegacy Laboratories (Shanghai) Co, Zhangjiang High-Tech Park Ltd, Building 7, 388 Jialilue Road, Shanghai, 201203 China; 5 Department of Human Genetics, David Geffen School of Medicine, University of California Los Angeles, Los Angeles, CA 90095 USA

**Keywords:** Non-small-cell lung cancer, Tumour heterogeneity

## Abstract

Lung adenocarcinoma (LUAD) harboring *EGFR* mutations prevails in Asian population. However, the inter-patient and intra-tumor heterogeneity has not been addressed at single-cell resolution. Here we performed single-cell RNA sequencing (scRNA-seq) of total 125,674 cells from seven stage-I/II LUAD samples harboring EGFR mutations and five tumor-adjacent lung tissues. We identified diverse cell types within the tumor microenvironment (TME) in which myeloid cells and T cells were the most abundant stromal cell types in tumors and adjacent lung tissues. Within tumors, accompanied by an increase in CD1C^+^ dendritic cells, the tumor-associated macrophages (TAMs) showed pro-tumoral functions without signature gene expression of defined M1 or M2 polarization. Tumor-infiltrating T cells mainly displayed exhausted and regulatory T-cell features. The adenocarcinoma cells can be categorized into different subtypes based on their gene expression signatures in distinct pathways such as hypoxia, glycolysis, cell metabolism, translation initiation, cell cycle, and antigen presentation. By performing pseudotime trajectory, we found that *ELF3* was among the most upregulated genes in more advanced tumor cells. In response to secretion of inflammatory cytokines (e.g., IL1B) from immune infiltrates, *ELF3* in tumor cells was upregulated to trigger the activation of PI3K/Akt/NF-κB pathway and elevated expression of proliferation and anti-apoptosis genes such as *BCL2L1* and *CCND1*. Taken together, our study revealed substantial heterogeneity within early-stage LUAD harboring *EGFR* mutations, implicating complex interactions among tumor cells, stromal cells and immune infiltrates in the TME.

## Introduction

Lung cancer is the leading cause of cancer-related deaths worldwide. Non-small cell lung cancer (NSCLC) accounts for ~85% of new lung cancer cases and has a poor 5-year survival rate below 16% [1]. In fact, lung adenocarcinoma (LUAD) becomes the most common pathological subtype of NSCLC, with increased frequency in young women and never smokers. Of note, *EGFR* mutations in LUAD are generally high in Asian women [2]. The most common *EGFR* mutations include deletions in exon 19 and L858R point mutation in exon 21. LUAD harboring these EGFR mutations is sensitive to tyrosine kinase inhibitor (TKI) treatment, but eventually develops acquired resistance after a year or so of progression-free period [3, 4].

Besides diverse pathological characteristics, NSCLC exhibits inter-patient and intra-tumoral heterogeneity in both tumor cells and microenvironments [5]. The tumor microenvironment (TME) consists of many cell types including immune infiltrates (e.g., mononuclear phagocytes, T cells, dendritic cells, B cells, and mast cells), cancer-associated fibroblasts (CAFs), and vascular endothelial cells. The TME components vary markedly among different tumors and play crucial roles in tumor initiation, progression, and metastasis [6, 7]. By modulating tumor-infiltrating immune cells, novel immunotherapies have already achieved great success in clinic. For instance, blocking the immune checkpoint molecules such as CTLA-4 and PD-1 can activate anti-tumoral cytotoxicity of T cells [8, 9]. Adoptive T-cell therapies using engineered T cells with chimeric antigen receptors (CARs) also hold great potential in clinical application [10]. However the efficacy of the immunotherapies is inconsistent among different patients, which may be resulted from heterogeneity of tumor cells and their microenvironments.

Recently, single-cell RNA-sequencing (scRNA-seq) technology has been used to study heterogeneous gene expression of different tissue samples. For example, droplet-based scRNA-seq methods with high throughput [11–15] have fueled the investigations of tumor microenvironment of many cancer types such as acute myeloid leukemia (AML) [12], breast cancer [16], pancreatic ductal adenocarcinoma [17], NSCLC [18]. In this study, we performed scRNAseq analysis of early-stage (stage I/II) LUAD from seven patients carrying EGFR mutations (Table 1). By comparing cellular heterogeneity of tumor cells with adjacent control lung tissues, we investigated the complex interactions among different cell types including tumor cells and other major cell components of the TME.

**Table 1:** Information of the patients for scRNA-seq.

Sample	Patient	Sex	Age	TNM stage	Pathological stage	Driver mutation	Smoking status	COPD	Metastases
T0418	LUAD1	F	48	T2aN0M0	IB	EGFR 19del	Never	No	No
T0422	LUAD2	M	61	T1bN1M0	IIA	EGFR L858R	Active	No	No
T0423	LUAD3	F	58	T1bN0M0	IA	EGFR 19del	Active	Yes	No
T0424	LUAD4	M	75	T1bN0M0	IA	EGFR 19del	Never	No	^a^
T0425	LUAD5	F	56	T2aN1M0	IIA	EGFR 19del	Never	No	No
T102	LUAD6	F	53	T1cN0M0	IA3	EGFR L858R	Never	No	No
S1028531	LUAD7	F	57	T2aN0M0	IB	EGFR 19del	Never	No	No

^a^Mediastinal and lymph nodes are swelling, and metastases are confirmed in clinical follow-up.

## Results

### scRNA-seq of seven LUAD samples harboring EGFR mutations

**Fig. 1:**
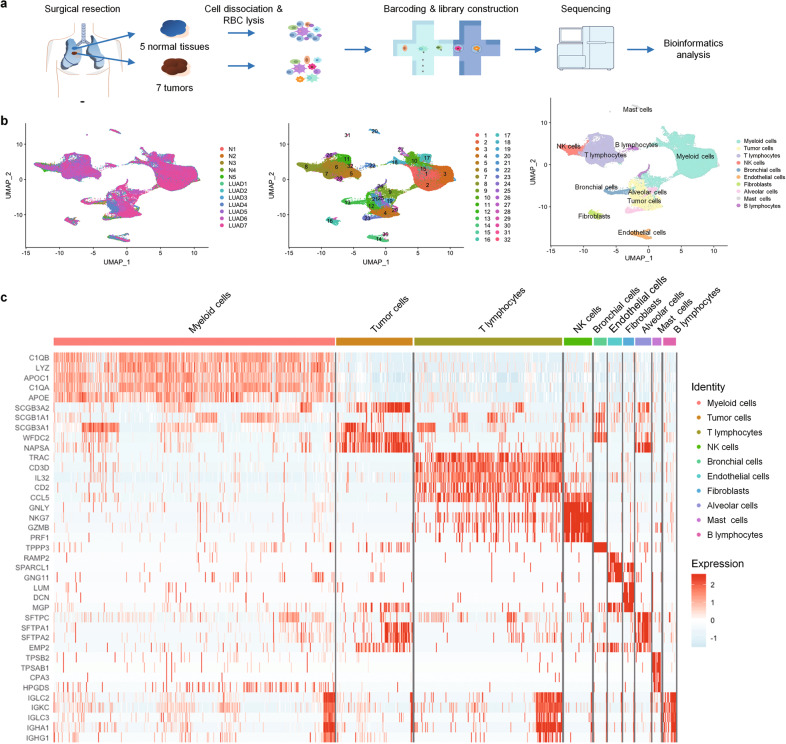
scRNA-seq of control tissues and LUAD samples harboring EGFR mutations. **a** Overview of the workflow. **b** UMAP plot of 125,674 profiled cells from all the samples (both LUAD tumor and tumor-adjacent normal tissues). Left: different color labeled for different samples. Middle: different color labeled for 32 clusters, respectively. Right: different color labeled for different cell types. **c** Heatmap of the top 5 DEGs (*p* < =0.05, fold change >=1.5) in each cell type.

We collected the resected tumor tissues from 7 untreated early-stage (stage I/II) LUAD patients (LUAD1 to 7) who all survived lobectomy surgery up to two years. All of them carried the most common *EGFR* active mutations, i.e., L858R point mutation and deletions in exon 19 (Table 1). For comparison, we also profiled cells from 5 tumor-adjacent normal lung tissues matching LUAD1-LUAD5, respectively. Immediately after resection, tumors and normal lung tissues were collected with enough aliquots for scRNA-seq and immunohistological evaluation. We then prepared single-cell suspensions from the tissues and constructed scRNA-seq libraries following 10X Genomics single-cell 3′ RNA library construction and sequencing pipeline (Fig. 1a). We obtained about 4.3 billion unique transcripts from 158,306 cells in which 1147 genes per cell were detected. After data processing and normalization, we obtained 125,674 cells for subsequent analysis (Supplementary Table S3) by removing potential doublets and the cells with poor quality (too few transcripts or too much mitochondria-derived RNA). We performed unsupervised clustering of the single cells from all seven tumor samples and five matched lung tissues and retrieved 32 distinct clusters (Fig. 1b) which were visualized by Uniform Manifold Approximation and Projection for Dimension Reduction (UMAP). To identify different cell types, we analyzed the expression of the canonical markers in each cluster (Supplementary Fig. S1, Supplementary Table S4) as well as the enrichment of differentially expressed genes (DEGs) (Fig. 1c), allowing us to categorize these clusters into tumor cells, bronchial/alveolar epithelial cells, myeloid cells, T lymphocytes, B lymphocytes, cancer-associated fibroblasts, endothelial cells and mast cells (Fig. 1b, c). The scRNA-seq data showed that myeloid cells and T lymphocytes accounted for the majority of the stromal cells, although the fractions of each cell type varied in different samples (Supplementary Fig. S2a). Consistently, immunohistochemistry also showed presence of many macrophages and T lymphocytes in tumor samples (Supplementary Fig. S2b).

### Tumor associate macrophages (TAMs) are in intermediate polarization states with pro-tumoral functions

**Fig. 2:**
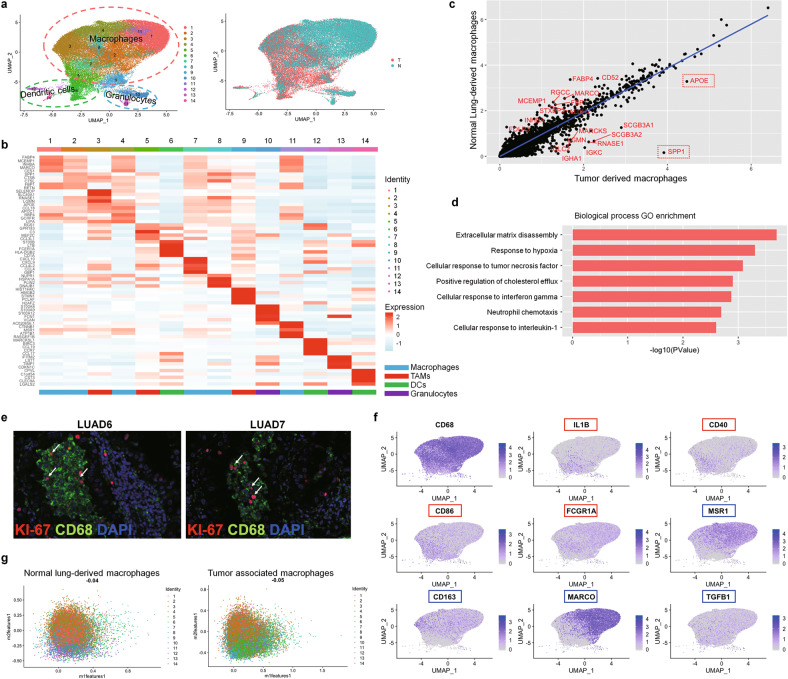
Tumor-associated macrophages (TAMs) exhibit pro-tumoral functions and are at intermediate polarization states. **a** UMAP plots with cells color-coded for 14 myeloid cell subclusters (left) and different tissue origins (right). **b** Heatmap showing the average expression of top 5 DEGs in each macrophage sub-cluster. **c** Scatter plot showing the top differentially expressed genes between tumor-associated macrophages and normal lung-derived macrophages. Red circle highlighted the two most highly expressed genes, *SPP1* and *APOE*, in tumor-associated macrophages. **d** Bar plot of representative enriched GO BP terms of DEGs in TAMs. **e** Immunostaining with anti- CD68 and KI67 antibodies identified the existence of proliferating macrophages in the LUAD tumor tissues. **f** UMAP showing the expression of canonical M1 (red) and M2 (blue) feature genes in all the macrophage (CD68-expressing) cells. **g** Scatterplots of normalized weighted mean expression of M1 and M2 signature genes (Supplementary Table S4) in each cell (dot) from all the macrophages derived from tumor samples (top) and normal lung tissues (bottom). The plots showing that TAMs and normal lung-derived macrophages express both M1 and M2 signature genes (Pearson correlation coefficient = 0.16), indicating they possess both polarized features and in various intermediate polarized states between M1 and M2 states.

Since the immune components could exert crucial influence on tumorigenesis and progression [19, 20], we first sought to investigate the features of the immune infiltrates in early-stage LUAD samples. As previously stated, myeloid cells account for the most abundant immune infiltrates in both tumors (~24%-70%) and normal lungs (~32%-64%) in our experiment (Supplementary Fig S2). We grouped all the myeloid cells into 14 heterogeneous subclusters and found that the majority (~90%) of the myeloid cells were CD68-expressing macrophages (Fig. 2a, b, Supplementary Fig. S3a). *CD11B* (*ITGAM*) was not highly expressed in the myeloid cells (data not shown). Increased dendritic cells and decreased granulocytes were found in the TME compared to the tumor-adjacent normal tissues (Fig. 2a, Supplementary Fig. S3). Compared to macrophages in the normal tissues, the TAMs have high expression of *APOE* (Fold change = 23.65909118, adjusted *p* value = 2.225074e−308) and *SPP1* (Fold change = 17.40700342, adjusted *p* value = 2.225074e−308) (Fig. 2c), which were reported to promote tumor cell growth and invasiveness [21, 22]. The DEGs enriched in TAMs were related to pathways such as extracellular matrix disassembly, response to hypoxia, positive regulation of cholesterol efflux and response to TNF and IL1B (Fig. 2d), implying that they may promote cell migration, angiogenesis [23], tumor progression [24] and inflammation. Interestingly, we identified a population of TAMs (Cluster 8 in Fig. 2b) which had high expression of cell-cycle related genes such as *NUPR1* (LogFoldChange = 1.102, adjusted *p* value = 2.225074e−308), *STMN1* (LogFoldChange = 1.69, adjusted *p* value = 2.225074e−308) and *MKI67* (LogFoldChange = 1.075131675, adjusted *p* value = 2.225074e−308). The existence of these proliferative macrophages was then verified by IHC using anti-CD68 and -KI67 antibodies (Fig. 2e).

Previous studies suggested that TAMs in tumors were phenotypically similar to anti-inflammatory M2-polarized macrophages [25]. To understand the polarization of TAMs in early-stage LUAD, we examined the expression of the conventional classic activated macrophage (M1) and alternative activated macrophage (M2) signature genes in these clusters (Fig. 2f, Supplementary Table S1). Interestingly, the macrophages in both tumors and normal lung tissues did not show exclusive M1 or M2 signature. Instead, they had similar expression level of both M1 and M2 signature genes (Fig. 2g). Taken together, while the TAMs in early-stage LUAD appear to express genes promoting tumorigenesis, they do not have distinct M1 and M2 polarization yet.

### Early-stage LUAD has immunosuppressive T lymphocytes and dendritic cell populations

**Fig. 3:**
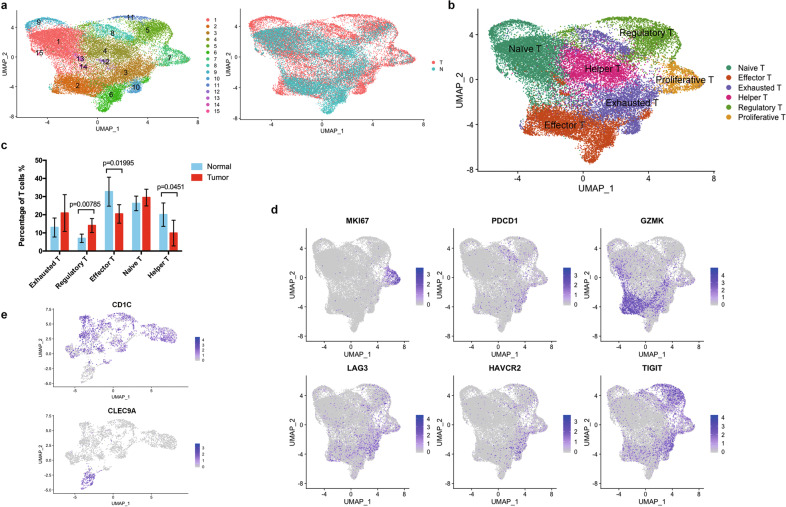
The TME of LUAD is enriched with regulatory and exhausted T cells accompanied by increased CD1C^+^ DCs. **a** UMAP plots of T cells derived from tumors and tumor-adjacent normal tissues with color-coded by different clusters (left) and different tissue origins (right). **b** UMAP plot of T cells derived from tumors and tumor-adjacent normal tissues with color-coded by different T-cell subtypes. **c** The proportions of T-cell subtypes in each LUAD sample and tumor-adjacent normal sample. **d** UMAP plots showing the expression signature of proliferating T cells in the TME, which express both effective and dysfunctional T-cell markers. **e** UMAP plots showing the expression of DC subtype markers, i.e., *CD1C* (CD1C^+^ DC) and *CLEC9A* (CD141^+^ DC), in all the DCs.

T cells account for ~20–60% of all the non-malignant cell populations (Supplementary Fig. S2). Non-biased clustering of all the T cells did not show any different clusters of T cells between the tumor samples and normal tissues (Fig. 3a). So we decided to check the expression of the T-cell subtype markers in each cluster. We found that the tumor-infiltrating T cells highly expressed regulatory and exhausted markers, such as *TIGIT* (LogFoldChange = 0.258224344, adjusted *p* value = 2.75e−143), *LAYN* (LogFoldChange = 0.071034001, adjusted *p* value = 1.90e−12)*, FOXP3* (LogFoldChange = 0.082017301, adjusted *p* value = 7.92e−13) and *CTLA4* (LogFoldChange = 0.101004531, adjusted *p* value = 4.63e−13), while the T cells in normal tissues highly expressed effector and naïve T-cell markers (Fig. 3b, c, Supplementary Fig. S4). We also identified a subtype of proliferative T cells (Cluster 7 with high KI67 expression) which displayed both effector T-cell features (e.g., GZMA) and dysfunctional markers such as LAG3, TIGIT, and PD-1, indicating compromised tumor cytotoxic activity (Fig. 3d).

The abundance of subtypes of T cells in the tumor microenvironment is strongly associated with prognosis in response to checkpoint blockade therapies [26]. T-cell infiltration and differentiation can be influenced by many intrinsic properties of the tumors such as local environment of cytokines, chemokines, and the presence of other immune cells including dendritic cells (DCs) in the TME [27]. There is a subset of tumor-derived macrophages highly expressing T lymphocyte chemotactic genes CXCL9, CXCL10, and CXCL11 (LogFoldChange = 0.4858127, 0.7869661, and 0.3042152 respectively; all adjusted *p* value = 2.225074e−308 vs. normal lung-derived myeloid cells; Supplementary Fig. S5). As known, DCs play a vital role in mediating T-cell chemotaxis, differentiation, and activation. In our dataset, we found that the DCs in LUAD were mainly CD1C^+^, implying their function in inhibition of effector T cells and promotion of regulatory T cells (Fig. 3d, e). All together, our results suggested that the early stage LUAD has an immunosuppressive TME, in which T cells differentiate towards the regulatory and exhausted subtypes accompanied by increased presence of CD1C^+^ DCs.

### Tumor cells within early-stage LUAD have distinct expression signatures

**Fig. 4:**
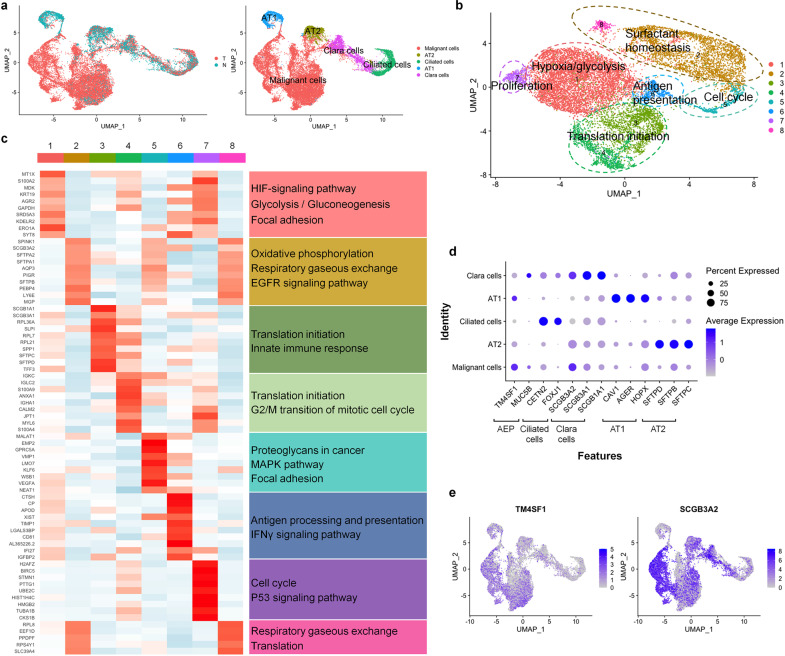
Tumor cells within early-stage LUAD have heterogeneous gene expression signatures. **a** UMAP plots of 20,802 malignant cells and normal lung epithelial cells from all the tumor and normal samples with color labeled for different tissue origins (left) and cell types (right), respectively. **b** UMAP plots of 14,456 malignant cells showed 8 distinct tumor cell clusters. They can be grouped based on the functional enrichment analysis of their DEGs. The tumor cells displayed expression signatures of hypoxia/glycolysis, oxidative phosphorylation, translation, cell cycle, antigen presentation and proliferation. **c** Heatmap showing the expression of DEGs in each tumor cluster and their enriched KEGG pathways. **d** Expression of canonical lung epithelial markers in each cell type. In a given cell identify, the sizes of circles indicate percentage of the cells expressing each marker gene; The shades of blue indicate average expression of each gene. **e** UMAP plots showing elevated *TM4SF1* and *SCGB3A2* expression in malignant cells than in normal lung epithelial cells.

To further verify our annotation of malignant and non-malignant cells in the tumor tissues, we inferred large-scale copy number variations (CNVs) from expression intensity of 15,414 genes (Supplementary Table S5) across each chromosome of potential malignant cells using the annotated lung epithelial cells in tumor-adjacent tissues as background control. The cells with aberrant CNVs were identified as malignant cells (Fig. 4a, Supplementary Fig. S6), which were further grouped into 8 clusters based on the functional enrichment analysis of their DEGs (Fig. 4b). These clusters were enriched in the pathways such as hypoxia, glycolysis, oxidative phosphorylation, translation initiation, cell cycle, and antigen presentation, respectively (Fig. 4c, Supplementary Fig. S7). In K-Ras-induced mouse LUAD, tumor cells could arise from AT2 cells in alveolar and Clara cells in the bronchioles [28, 29]. However, we did not see high expression of the classic AT2 cell marker *SFTPC* or Clara cell marker *SCGB1A1* in the tumor cell, except in a population of cluster 3 (Supplementary Fig. S8). We then examined the expression of conanical markers in tumor cells compared to the normal lung epithelial cells. We found that the Clara cell progenitor marker *SCGB3A2* [30, 31] (LogFoldChange = 16.21089, adjusted *p* value = 9.088149e−93) and alveolar epithelial progenitor (AEP) marker *TM4SF1* [32] (LogFoldChange = 0.9938625, adjusted *p* value = 1.553989e−104) were upregulated in tumor cells (Fig. 4d, e, Supplmentary Fig. S9). As they highly expressed both proximal and distal epithelial progenitor markers, the tumor cells were composed of heterogeneous populations with expression profiles of diverse cell lineages.

### Pseudotime trajectory analysis shows that *ELF3* is upregulated in more advanced tumor cells

**Fig. 5:**
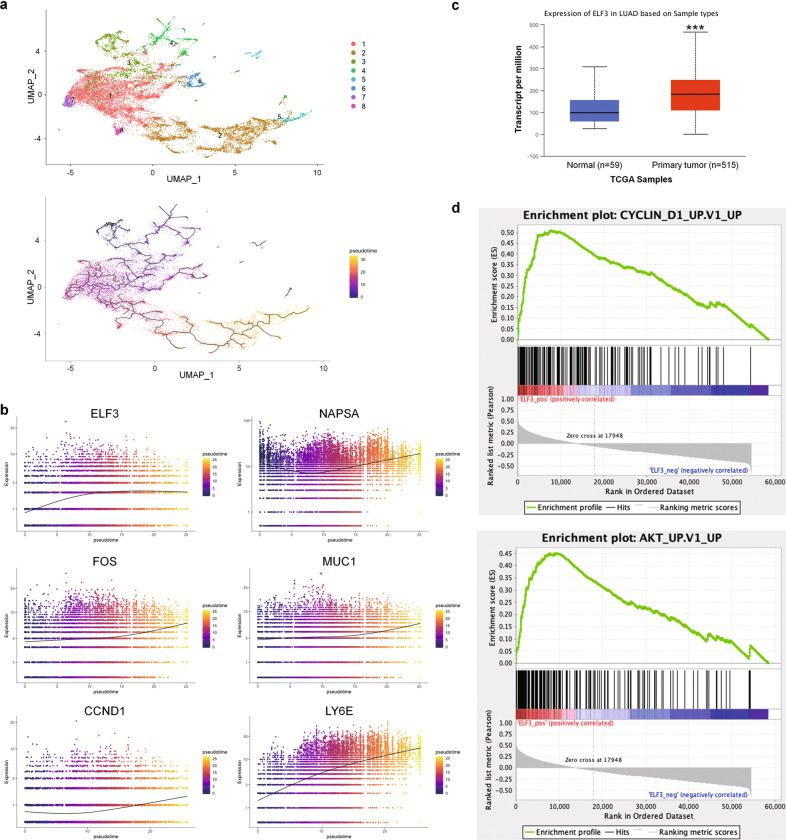
Pseudotime trajectory analysis revealed the upregulated genes in more advanced tumor cells. **a** Pseudotime trajectory of all the malignant cells derived from 7 LUADs. Top: pseudotime trajectory tree of all the tumor cells with different colors indicating different clusters identified by Seurat. Bottom: all the tumor cells were colored by their assigned pseudotime values. **b** Jitter plots showing the expression level of the genes changing with pseudotime. *ELF3*, *FOS, MUC1, CCND1,* and *LY6E* were among the most significantly upregulated genes in cells with larger pseudotime. NAPSA, a LUAD prognostic marker, also have higher expression in more advanced tumor cells. **c** The TCGA LUAD (*n* = 515) dataset showing ELF3 was upregulated in LUAD samples compared to the normal lung tissues (*n* = 59). **d** Gene set enrichment analysis (GSEA) showing that the CCND1 and AKT upregulation gene sets were overrepresented in the tumors with high *ELF3* level, indicating *ELF3* level was associated with tumor cell proliferation and progression.

As described above, our results revealed different tumor cell clusters with DEGs enriched in distinct pathways, implying progressional heterogeneity among them. We then asked if the single-cell transcriptome could gain temporal information of differentiation states during LUAD progression. We pooled all the malignant cells (Fig. 1b) and constructed a single-cell pseudotime trajectory using monocle3 R package. Monocle3 did a dimension reduction on all the tumor cells and ordered the cells based on their progression states. We manually set the root-state of the pseudotime trajectory within the cells of cluster 3 because they still expressed normal lung epithelium markers such as alveolar type 2 (AT2) cell marker SFTPC and Clara cell marker SCGB1A1, suggesting that they still remain the characteristics of normal lung epithelial cells (Supplementary Fig. S8). Then the pseudotime trajectory was obtained after calculating the pseudotime value of each cell (Fig. 5a). Using graph-test function of monocle3, we identified the genes upregulated in more advanced cells within the pseudotime trajectory (Supplementary Table S6). Although they were on the top of the list, long non-coding RNAs (LncRNAs) MALAT1 [33] and NEAT1 [34] have already been characterized as the hallmarks of metastasis in lung cancer [35] and other malignant tumors [36, 37]. We noted that additional altered genes encode structural proteins such as membrane channels (e.g., APQ3) and transporters (e.g., SLC34A2 and NPC2) that may be coupled to key regulators in tumor cells. We were particularly interested in studying those genes that are associated with cancer progression, TME signals, and poor prognosis such as LY6E [38], NAPSA [39], MUC1 [40, 41], ELF3 [42, 43], and FOS [44] (Fig. 5b). Some of them have been well characterized in lung cancers. For example, NAPSA is a common prognostic marker for LUAD. LY6E [38], MUC1 [45], and FOS [46] play important roles in immune escape and suppressive immune microenvironment in various types of cancers. In this study, we decided to focus on the role of ELF3, a transcriptional factor regulating lung epithelial development [47], which is implicated in airway inflammation [48] and mediates inflammatory signal and tumor progression in prostate cancer [49] and colorectal cancer [50].

To address the role of ELF3 in LUAD, we used the TCGA LUAD data (*n* = 515) and found that ELF3 was upregulated in tumor samples compared to the normal lung tissues (Fig. 5c). In addition, the gene set enrichment analysis (GSEA) of the TCGA dataset showed that the CCND1 and AKT upregulated gene sets were overrepresented in the tumors with higher *ELF3* expression level (Fig. 5d). In conclusion, these results suggested that *ELF3* was upregulated in more advanced LUAD cells and associated with upregulation of CCND1 and AKT in LUAD.

### ELF3 promotes tumor growth through PI3K/AKT/NF-κB signaling pathway in LUAD cells after IL1B induction

**Fig. 6:**
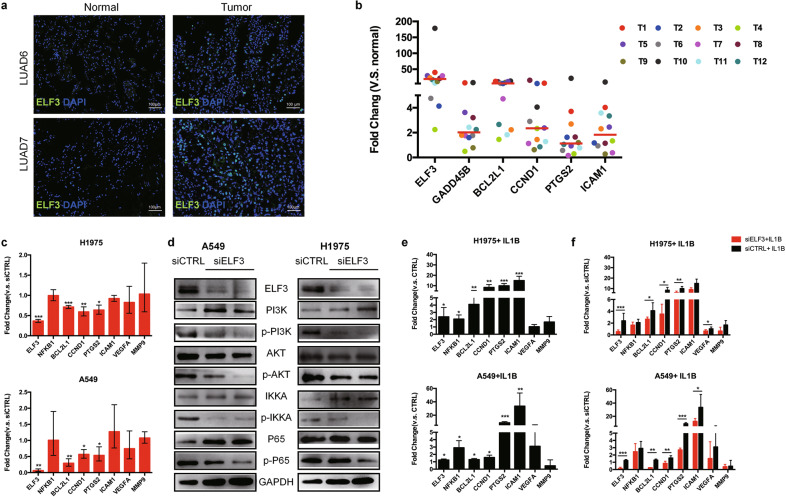
ELF3 can be induced by IL1B and promotes tumor growth through PI3K/AKT/NF-κB pathway. **a** Representative immunostaining showed that ELF3 expression in LUAD6 and LUAD7 is higher than that in the matched normal lung tissues. **b** Expression of ELF3 and selected NF-κB target genes in additional 12 LUAD tumor samples by qRT-PCR. The plot showed that the median expression of ELF3 and NF-κB targeting genes related to proliferation and anti-apoptosis are higher in the tumor tissues than their matched normal lung tissues. Colored dots refer to different individuals. **c** Expression of ELF3, NFKB1, and selected NF-κB target genes in NCI-H1975 (top) and A549 (bottom) cell lines by qRT-PCR after knockdown of ELF3 by siRNA (The controls were transfected with control siRNA (siCTRL)). In both cell lines, downregulation of ELF3 results in decreased expression of BCL2L1, CCND1, and PTGS2 which are responsible for anti-apoptosis, proliferation, and inflammation in tumor cells respectively. **d** Western blot showing the protein and phosphorylation levels of the key components in PI3K/AKT/NF-κB pathway in NCI-H1975 and A549 cells transfected with siELF3 or siCTRL. The phosphorylation levels of PI3K, AKT, IKKa, and P65 were decreased after ELF3 knockdown with unaltered total protein levels. **e** qRT-PCR showing the expression levels of *ELF3*, *NFKB1,* and selected NF-κB target genes in NCI-H1975 (top) and A549 cells (bottom) treated with 10 ng/ml IL1B for 1 h. The plots showed that the expression of *ELF3* and NF-κB target genes such as *BCL2L1*, *CCND1*, *PTGS2,* and *ICAM1* are increased in both cell lines after IL1B treatment. **f** qRT-PCR showing expression of *ELF3*, *NFKB1,* and the NF-κB target genes in NCI-H1975 (top) and A549 cells (bottom) which were transfected with siELF3 or siCTRL before being treated with 10 ng/ml IL1B for 1 h. The plots showed that IL1B induced upregulation of NF-KB target genes are compromised by ELF3 knockdown in both cell lines.

ELF3 can regulate cell cycle and proliferation in NSCLC [42] and chemical-induced lung injury [51]. High expression of *ELF3* also favors tumor growth through activation of MAPK and NF-κB pathway [49, 52]. Given that NF-κB pathway plays essential roles in activating survival genes for cancer cells as well as inflammatory response in immune cells [53, 54], we speculate that ELF3 may function as an important modulator of NF-κB pathway in LUAD. We first confirmed the upregulation of ELF3 at protein level in the tumor tissues compared to the normal lung tissues (Fig. 6a). We then examined the expression of *ELF3* and NF-κB target genes in additional 12 primary LUAD tissues (T1-T12, Supplementary Table S7) by quantitative real time-PCR (qRT-PCR). We found that *ELF3* was highly expressed in all the tumor tissues compared to the matched normal samples. Some NF-κB target genes such as *CCND1*, *BCL2L1* and *GADD45B* and *ICAM1* which are responsible for proliferation, anti-apoptosis and adhesion were also upregulated in the tumor tissues (Fig. 6b).

Since other cell types in TME, especially immune cells, also highly express NF-κB target genes, we decided to use LUAD cell lines A549 and NCI-H1975 to further investigate the function of ELF3 in tumor cells. After knocking down *ELF3* by siRNAs, the expression of *NFKB1* remained unchanged (Fig. 6c). Some NF-κB target genes such as *BCL2L1*, *CCND1,* and *PTGS2* were downregulated in both cell lines, while others responsible for angiogenesis (i.e., *VEGFA*) and metastasis (i.e., *MMP9*) remained unchanged (Fig. 6c). The reduced gene expression was resulted, at least in part, from the inactivation of PI3K/AKT/NF-κB pathway, as the phosphorylated proteins of PI3K, AKT, IKKa and P65 were all decreased after *ELF3* knockdown (Fig. 6d).

It is known that pro-inflammatory cytokines secreted by immune cells such as IL1B and TNFɑ can activate NF-κB pathway in different cell types [55–57]. To determine whether ELF3 is involved the activation of NF-κB pathway in LUAD cells, we first treated A549 and NCI-H1975 cells with IL1B. We found elevated expression of *ELF3* and the NF-κB target genes related to cell survival and inflammation such as *BCL2L1*, *CCND1*, *PTGS2,* and *ICAM1* (Fig. 6e), which was compromised when *ELF3* was knocked down in both cell lines (Fig. 6f). It should be noted that A549 and NCI-H1975 cells do not have *CCND1* amplification according to the COSMIC cell line project database [58]. *CCND1* amplification is seen in ~3.64% of the TCGA LUAD cohort. We inferred some copy number gains of *CCND1* on chromosome 11 (Supplementary Fig. S6c), which may contribute to its overexpression in some tumor cells in addition to transcriptional regulation mediated by ELF3. Taken together, pro-inflammatory cytokine IL1B in the tumor microenvironment can upregulate ELF3 in LUAD tumor cells, which augments the activation of NF-κB pathway to favor tumor cell survival and growth. Our results implicated that ELF3 in LUAD tumor cells may serve as a novel therapeutic target to prevent tumor growth.

## Discussion

The crosstalk with tumor cells and tumor microenvironment could promote tumor progression and metastases. However, few of the previous studies have investigated the transcriptome of lung tumor cells and their TME together, either in bulk or at single-cell level [18, 59–62]. Using droplet-based scRNA-seq technology (10X genomics), we analyzed different cell subpopulations in tumor samples simultaneously and comprehensively. We identified distinct subpopulations of tumor cells and their TME components, indicating that the heterogeneity in LUAD has already emerged at its early stage of tumorigenesis.

The immunal TME possesses either pro- or anti-tumor properties and has been widely studied for potential immunotherapy [63, 64]. We found that myeloid cells and T cells are the most abundant stromal cell types in the early-stage LUAD samples and their adjacent control tissues (Supplementary Fig. S2a). It is well known that alveolar macrophages, which do not express CD11b, are the most abundant cell population of resident immune cells [65]. Consistenly, at single-cell resolution, we show that lung resident macrophages are the most abundant stromal cell population, exhibiting high expression of *CD68* but not *CD11B* (Fig. 2, Supplementary Figs. S2 and 3). Our results suggest that the overall immune TME is promoting tumor and immunosuppressive. Indeed, TAMs show high levels of gene expression enriched in the pathways which promote cell migration, angiogenesis, tumor progression, and inflammation (e.g., *APOE* and *SPP1*) (Fig. 2). Finally, the immunosuppressive state of TAMs in LUAD cannot be simply explained by M2 polarization, which is consistent with the previous reports that TAMs are not polarized to a distinct state of either M1 or M2 [16, 66].

Interestingly, we identified proliferating cell subtypes in both TAMs and T cells, which are characterized by high expression of proliferation marker *MKI67*. Although they have barely been reported in LUAD, proliferating TAMs and T cells were investigated in other tumor types. In breast cancer, proliferating TAMs were identified by proliferating cell nuclear antigen (PCNA) and found to be associated with tumor progression and poor prognosis [67, 68]. These PCNA^+^ proliferating TAMs in breast cancer also had high expression of *MKI67* [69]. In the T cells, we identified a proliferating subtype that simultaneously express both effector T-cell and dysfunctional markers (Fig. 3c). A recent research reported a highly proliferating population of dysfunctional T cell in the TME of human melanoma [70]. We also found more regulatory and exhausted T-cell clusters in the TME of LUAD, correlated with increased CD1C^+^ DC population.

We investigated the role of an important modulator ELF3 in mediating interaction between TAMs and tumor cells in LUAD. ELF3 is an E26 transformation-specific (ETS) transcription factor highly expressed in epithelial-rich tissues. Its expression is relatively low in adult lung compared to fetal lung [71]. *ELF3* was initially found to be overexpressed in HER2 positive breast cancer [72]. Soon after, its high expression was also reported in LUAD primary tumor and cell line A549 [71]. ELF3 is associated with poor prognosis in different tumor types including LUAD [42, 43], colorectal cancer [73], and hepatocellular carcinoma [74]. In prostate cancer, ELF3 regulates the NF-κB pathway after stimulated by inflammatory signals [49], suggesting that it may connect the TME signals and tumor progression. We demonstrate that some TAMs highly express inflammatory cytokine genes such as *IL1B* (Fig. 2f) that activates NF-κB pathway. We hypothesize that IL1B released from TAMs triggers elevated expression of *ELF3* in LUAD tumor cells. Knocking down ELF3 expression attenuates activation of NF-κB pathway genes in two LUAD cell lines A549 (KRAS mutation) and NCI-H1975 (EGFR mutation L858R), supporting the conclusion that inflammatory cytokines (e.g. IL1B) can regulate tumor cell proliferation and anti-apoptosis through *ELF3* action.

Taken together, this work provides a valuable resource in understanding the heterogeneity and immunal cell profile in the early-stage LUAD harboring EGFR mutations in Asian patients. We identify crucial genes including *ELF3* in mediating the interactions among tumor cells and their TME components, suggesting that ELF3 could be a therapeutic target in LUAD for future drug discovery.

## Material and methods

### Preparation of single-cell suspensions from biopsies

The tumor tissues and tumor-adjacent non-malignant lung tissues (~1.5 cm × 1 cm × 0.5 cm) were resected after the surgeries and transported in DMEM/F12 medium (Gibco 11320082) on ice to the research facility within an hour. The details of tissue collection and dissociation protocol are described in Supplementary Materials and Methods.

### Single-cell RNA library construction, sequencing, and data analysis

Single-cell suspensions (~1 × 10^6^ /ml) were submitted to 10X genomics Chromium Controller to generate single-cell GEMs (gel beads in emulsion). The constructed Chromium Single Cell 3’ Library aiming for estimated 6000 cells for one sample following the manufacture’s instruction (10x genomics Chromium Single Cell 3′ Library & Gel Bead Kit v2). Then libraries were sequenced by Illumina Hiseq X Ten platform. The raw sequence data reported in this paper have been deposited in the Genome Sequence Archive [75] in BIG Data Center [76], Beijing Institute of Genomics (BIG), Chinese Academy of Sciences. The access numbers are CRA001477 and CRA001963. Details of bioinformatics analysis of RNAseq data are provided in Supplemental Materials and Methods.

### Expression of M1 and M2 signature genes

M1 and M2 signature genes (Supplementary Table S1) were curated form literature. The normalized weighted mean expression of those genes was calculated by Seurat’s AddModuleScore and their correlation was suggested by Pearson correlation coefficient.

### GSEA analysis

LUAD fpkm expression dataset (*n* = 585) and its phenotype labels were downloaded from UCSC Xena (https://xenabrowser.net). GSEA analysis was done by GSEA software [77] and gene set used was c2 gene set from Molecular Signatures Database (MSigDB).

### Immunostaining

All the dissected tissues were fixed with 4% PFA and embedded with paraffin. The 4 µm tissue sections were cut and stained with hematoxylin and eosin for histology inspection. For immunohistolochemistry, anti-human CD3 (Servicebio GB11014), anti-human CD68 (Zsbio zm0060) were used. The immunofluorescence staining was performed as routine. The antibodies used were anti- human ELF3 (ABclonal A6371), anti-human CD68 (ZSGB ZM-0060), anti-human KI67 (Servicebio GB14102), anti-human SFTPC (ABclonal A1835), anti-human SCGB3A1 (R&D Systems MAB27901-SP) and anti-human SCGB3A2 (Abcam ab181853). The images were captured by Nikon ECLIPSE C1.

### Cell culture, shRNA interference, and IL1B treatment

A549 and NCI-H1975 cells were cultured in RPMI-1640 medium (ThermoFisher Scientific 11875119) supplied with 10% FBS (ThermoFisher Scientific 10099141) and 1% penicillin and strepmycin (ThermoFisher Scientific 15140163) in 5% CO_2_ at 37 ^o^C. The siELF3 and siControl RNAs were pre-designed purchased from Thermofisher Technologies (4392420) and transfected using RNAiMAX transfection reagent (ThermoFisher Scientific 13778030) following the manufacturer’s instruction. For IL1B treatment of both cell lines, recombinant human IL1B powder (R&D systems 201-LB-010) was diluted with PBS + 1% FBS to 10 mg/µl. 20 µg of (20 µl) IL1B were added to 2 ml cultured medium of each well of six-well plate. After IL1B treatment for 1 h, total RNA and protein of the cells were isolated for subsequent analysis.

### qRT-PCR

The total RNAs were extracted by TRIzol reagent (Invitrogen 15596026). The reverse transcription was done using RevertAid RT Reverse Transcription Kit (ThermoFisher Scientific K1691) following the manufacture’s protocol. The qPCR primers (Supplementary Table S2) were designed on https://sg.idtdna.com. The qPCR amplification was done using Taq polymerase (Takara RR820A) on Roche LightCycler 480 instrument. Fold change was calculated as 2^(-△△CT)^; △△CT = △CT(sample)- △CT(control); △CT = CT (gene)-CT (internal reference(B2M)).

### Western blotting

The cells were lysed by RIPA lysis buffer (Epizyme PC101) followed by centrifuging at 12,000 × *g* for 5 min at 4 ^o^C. Total protein was collected and stored in −80 ^o^C until use. Western blotting was performed as routine. The primary antibodies used were anti-human ELF3 (ABclonal A6371), anti-human PI3K (Cell Signalling Technology 4249), anti-human P-PI3K (Cell Signalling Technology 4228), anti-human AKT (ABclonal A11030), anti-human P-AKT (Cell Signalling Technology 4060), anti-human P65 (Abcam ab16502), anti-human P-P65 (Abcam ab86299), anti-human IKKA (ABclonal A0422) and anti-human P-IKKA (ABclonal AP0546). Secondary antibody was goat anti-rabbit IgG (H + L) HRP (ABways AB0101).

## Web resources

The raw sequence data reported in this paper have been deposited in the Genome Sequence Archive in BIG Data Center, Beijing Institute of Genomics (BIG), Chinese Academy of Sciences. They are publicly accessible at https://bigd.big.ac.cn/gsa.

## Supplementary information

Supplementary information

Supplementary Table S5

Supplementary Table S6
