# Supplementary material for: Single-cell RNA sequencing reveals heterogeneous tumor and immune cell populations in early-stage lung adenocarcinomas harboring EGFR mutations
Source: Oncogene. 2020 Nov 3;40(2):355–68. doi: 10.1038/s41388-020-01528-0 (PMC7808940; doi:10.1038/s41388-020-01528-0)
Supplement: Supplementary file 1 — Supplementary information [file 41388_2020_1528_MOESM1_ESM.docx]

**Supplemental Data**

**Supplemental Materials and Methods**

**Patients**

This study was approved by the ethics committee of Shanghai Pulmonary Hospital. We complied with all the relevant ethical regulations. The patients recruited in this study provided written informed consent for sample collection and data analyses. Only the patients who had untreated, primary non-metastatic LUAD that underwent tumor resection with curative intent were enrolled in this study.

**Details in the Preparation of single cell suspensions**

The tumor tissues and tumor adjacent non-malignant lung tissues were rinsed with cold PBS (Hyclone SH30256.01) to wash out the external blood and dead cells. Then they were cut into three pieces. One piece was snap frozen by liquid nitrogen, while one was immersed in 4% PFA for subsequent immunostaining. The other was for scRNA-seq, which was put in 1 ml cooled PBS and minced to small pieces (smaller than 1 mm^3^) within 5 min. Then the tissues were transferred into 10 ml digestion buffer (DMEM/F12 Medium+1% FBS (Gibco 10099-141) + 2 mg/ml of collagenase II （Gibco 17101015) + 10 IU/µl DNase I (Roche 11284932001)) and incubated in 37^o^C for 15 min with manual shaking every 5 min. The samples were vortexed for 10 s, pipetted up and down for 1 min using pipettes of different sizes (25 ml, 10 ml and 5 ml), and passed through a 70 um cell strainer (Corning 431751). The passing through was centrifuged with 120X g for 6 min to collect the single cells. The cells were resuspended with 2 ml RBC lysis buffer (BD Biosciences 555899) in RT for 10 min to remove the red blood cells. The cell suspension was centrifuged at 120X g for 5 min. The cell pellets were finally resuspended with 1ml cooled PBS before microscopic inspection and scRNA-seq library construction.

**Details of scRNA-seq data analysis and graphing**

After we obtained raw sequence data, we mapped sequences to the human genome reference (GRCh38) using CellRanger (3.0.1) pipeline. The generated gene-barcode matrixes were submitted to Scrublet ^21^ to remove the potential doublets. Then the 10x data matrixes were imported into Seurat V3.0 R package (https://satijalab.org/seurat) to perform data filtration, sample integration, gene normalization, dimension reduction and data visualization. All the samples were integrated as one object by Seurat “IntegrateData” function. Cells with low feature counts (<200) and high percent of mitochondrial genes (>10%) were removed. Dimension reduction was done by Seurat “RunPCA” function. Then Uniform Manifold Approximation and Projection for Dimension Reduction (UMAP) was used to visualize single-cell clusters, by graph-based clustering the cells, employing the top 30 principle components with the largest variance (at resolution=0.5 for all the merged samples, and resolution=0.8 for subclustering each cell type).

Based on the cell clustering results, likelihood ration statistic test was used to screen differentially expressed genes (DEGs) of each cluster by Seurat’s Bimod. The genes can be considered as DEGs when they have the expression that satisfy adjusted p<0.05 (corrected P value from T test by Benjamini-Hochberg correction) and LogFoldChang>=0.585 compared to other clusters. Cluster specific maker genes were chosen by their significantly up-regulation in one cluster but not in other clusters. DEGs between tumor-derived cells and tumor-adjacent normal lung-derived cells were detected by Seurat’s “FindMarkers” function using Wilcoxon Rank Sum test.

**Annotation of each cluster**

First each cluster was annotated based on the expression of the canonical marker genes that we have curated from literatures (Supplementary Table S4). We also referred cluster specific DEGs that are known to be cell markers to help verify the cell type.

**CNV inferred from scRNA-seq**

InferCNV (https://github.com/broadinstitute/inferCNV) was used to identify large-scale chromosomal copy number variations of potential malignant cells using single cell RNA-seq data. The expression intensity of 15,414 genes (Supplementary Table S5) across the malignant cell genome was compared to annotated non-malignant lung epithelial cells. The heatmap was generated to illustrate the gains and deletions of large segments of chromosomes in the malignant cell genome compared to the non-malignant epithelial cells in all the samples.

**Cell pseudotime trajectory analysis**

Monocle 3 (https://cole-trapnell-lab.github.io/monocle3) was used to construct the pseudotime trajectory. In detail, the “cell_data_set” was built from the Seurat object of all the tumor cells using the data slot of the integrated assay. Dimension reduction was done by UMAP, “learn_graph” and “order_cells” functions were used to establish the trajectory. Each single cell was projected on the tree and formed the trajectory by DDRThree. The “root_state” of this trajectory tree was manually appointed in cells of cluster 3, because they still express normal AT2 cell marker *SFTPC* and Clara cell marker *SCGB1A1*, suggesting that they still remain the normal lung epithelial characteristics.

**Identification of upregulated DEGs in advanced tumor cells**

Genes with various expression over the trajectory were detected by “graph_test” function of monocle 3. The DEGs in cells over the trajectory (Moran’s I > 0.2 and q < 0.01) were selected for subsequent analysis (Mitochondrial genes and house keeping genes related to ribosomal RNA transcription and production were excluded.). Before referred to as upregulated genes in cells of advanced progressional states (Supplementary table S5), these DEGs were further filtered so that their expression is higher (LogFoldChange>0.3, adjusted p<0.05) in more aggressive tumor cells (cluster 2 and 5 in Fig 5A).

**GO biological process term and KEGG pathway analysis**

Enriched Gene Ontology (GO) biological process terms and Enriched Kyoto Encyclopedia of Genes and Genomes (KEGG) pathways of the DEGs were identified using the Database for Annotation, Visualization and Integrated Discovery (DAVID) online tool ( https://david.ncifcrf.gov/summary.jsp) and ClusterProfiler R package ^22^. The lists of DEGs that satisfy adjusted p value<0.05 and LogFoldChange>=0.25 in a cluster were used, then we chose representative GO terms and KEGG pathways that satisfy p<0.05 and illustrated by ggplot2.

**Supplementary Figures S1 to S9**


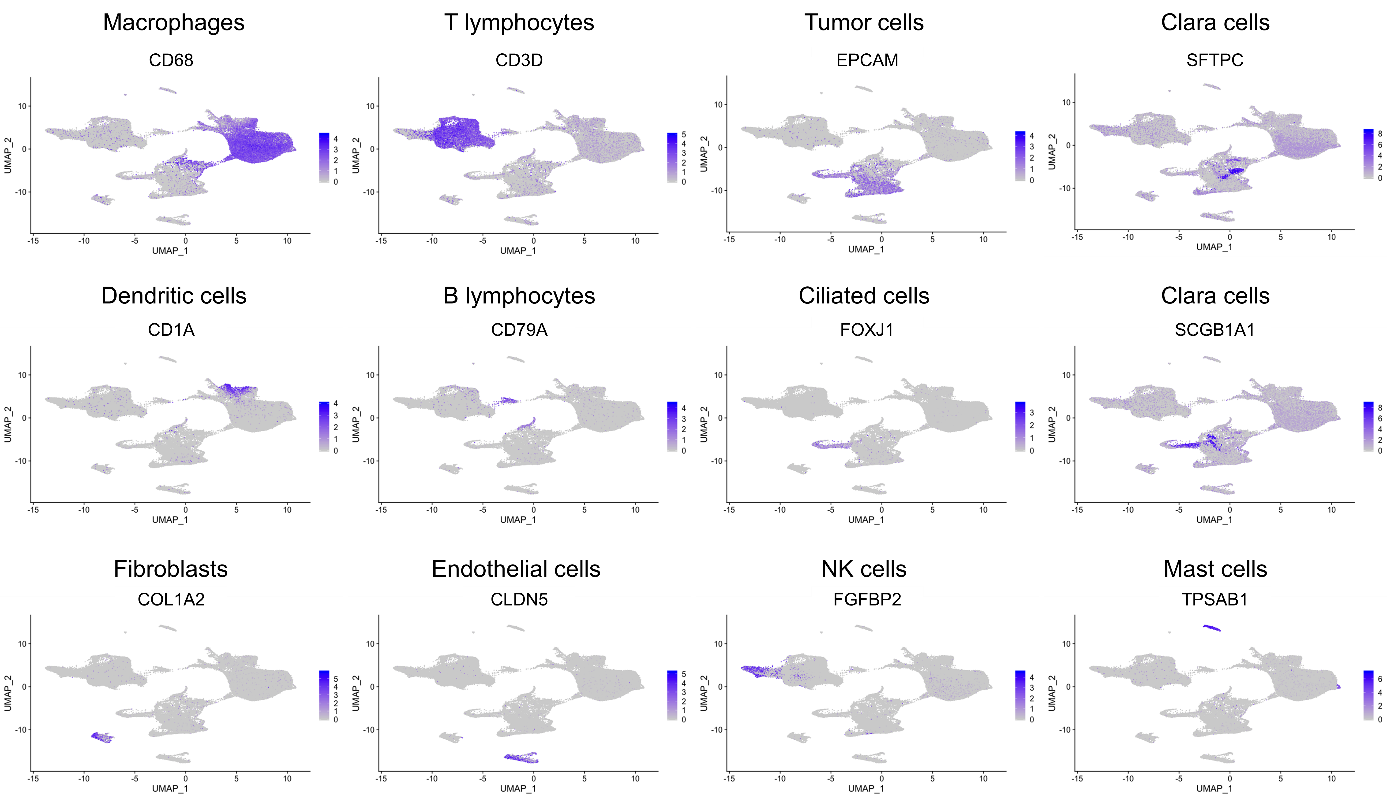


**Figure S1 Expression of cell-type markers in all the cells profiled by scRNA-seq**

UMAP plots showing the marker gene expression of different cell types including malignant cells, myeloid cells, T lymphocytes, B lymphocytes, normal lung epithelial cells, cancer associated fibroblasts (CAFs) and endothelial cells.


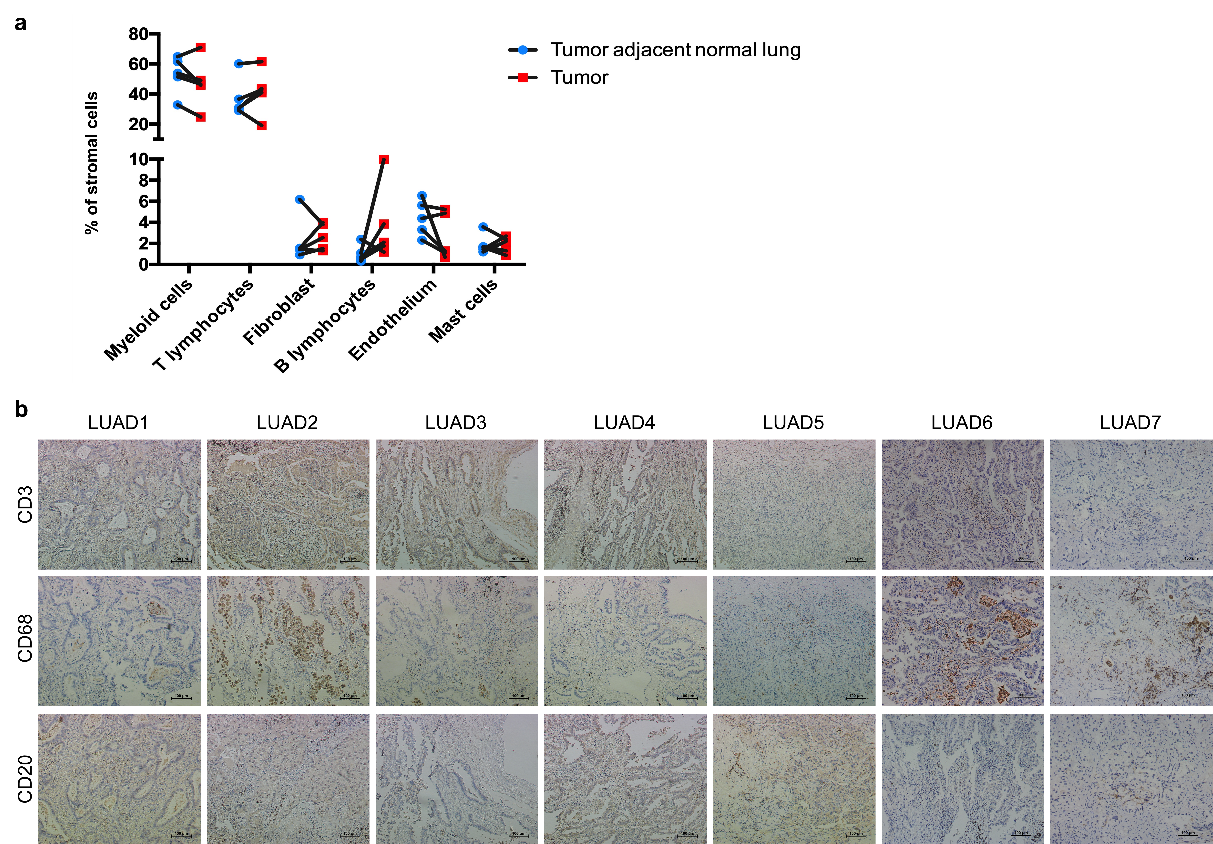


**Figure S2 Characteristics of the tumor tissues**

(a) Dot plot showing proportions of stromal cells in tumors and tumor-adjacent lung tissues. The plot showed myeloid cells and T lymphocytes were the two most abundant stromal cell populations in both tumor and normal lung tissues. However, the percentage of each stromal cell type varied among different patients.

(b) Immunohistochemical staining of CD3, CD68 and CD20 in 7 LUAD samples, indicating T lymphocyte, macrophages and B lymphocyte infiltration in each LUAD sample respectively.


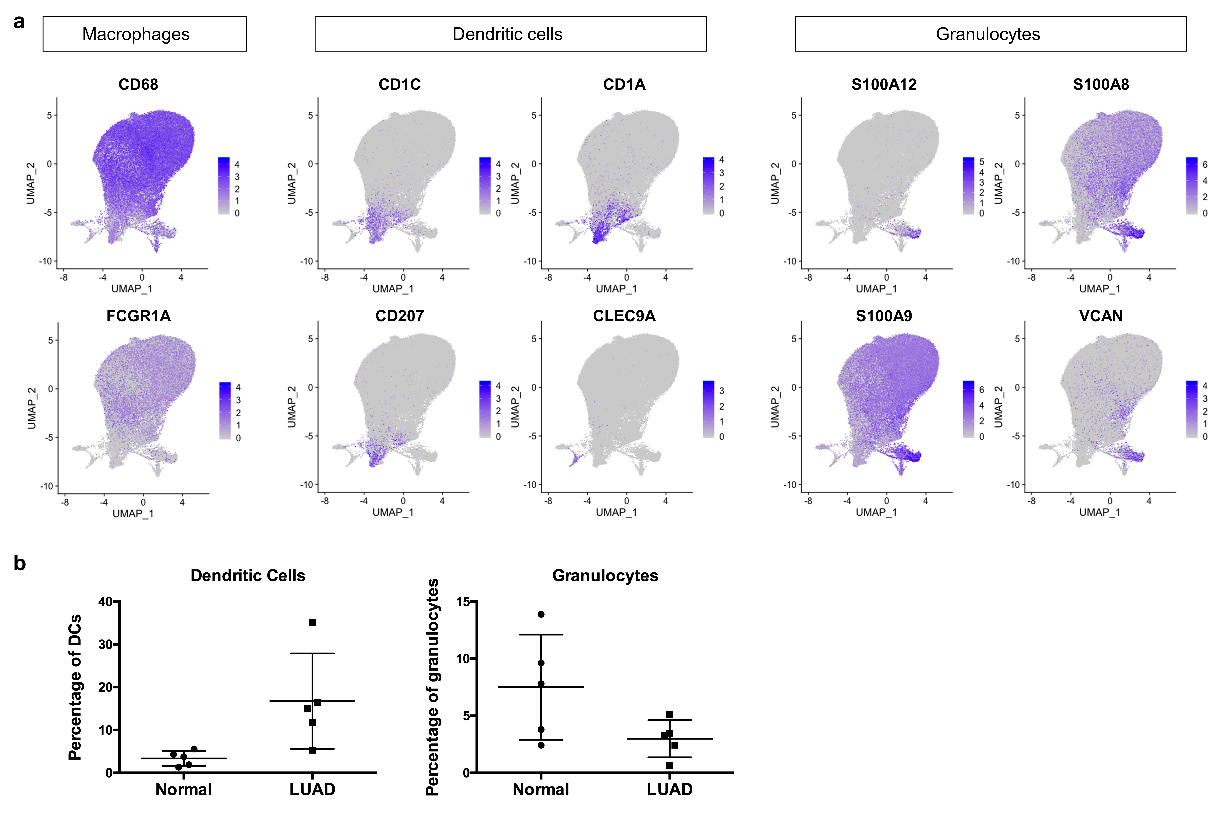


**Figure S3 Myeloid subsets in LUADs and tumor-adjacent lung tissues**

(a) UMAP plots showing the expression of macrophage, dendritic cell and granulocyte markers in all the myeloid cells.

(b) The plots showing that the percentage of DCs (dendritic cells divided by all the myeloid cells) is increased in each LUAD tissue compared to the tumor adjacent lung tissue (p value= 0.0294), while that of the granulocytes is decreased (p value= 0.0726). The lines indicate means with SD.

**
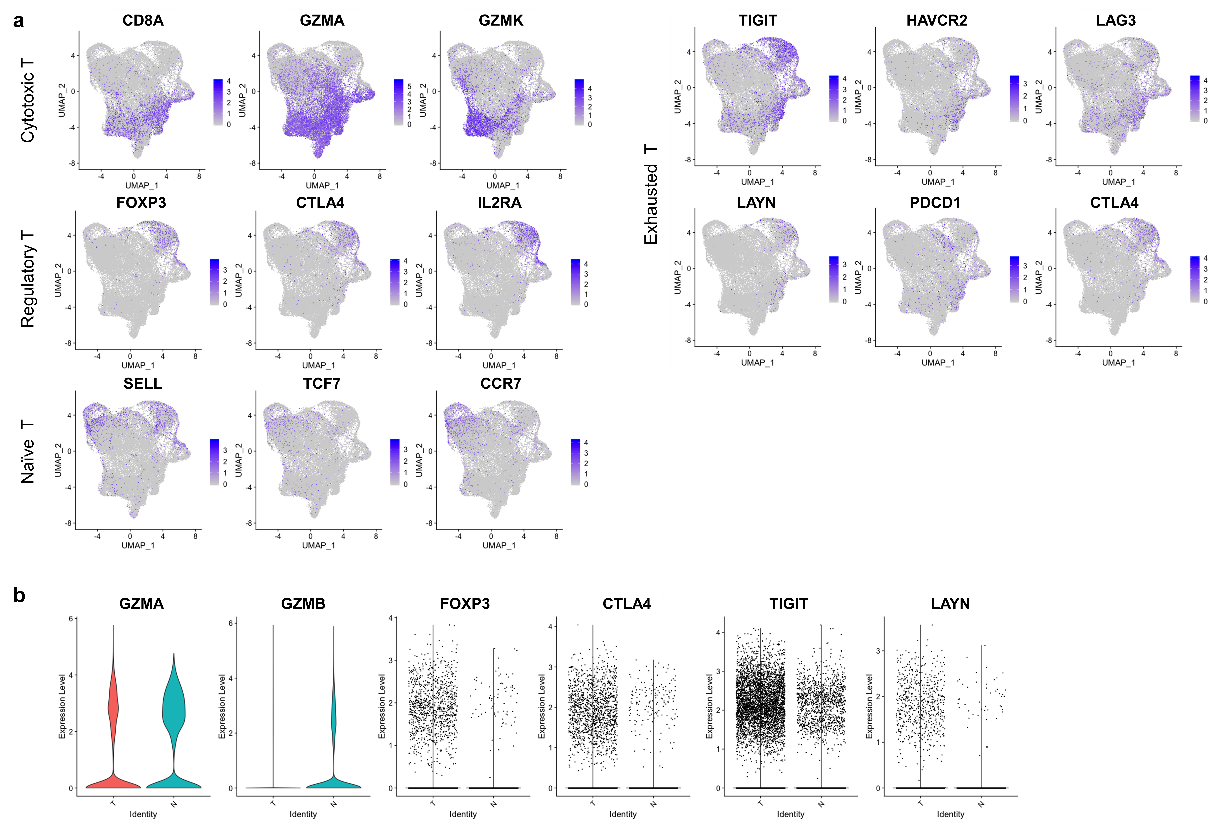
**

**Figure S4 Expression of T lymphocyte subtype markers in all the T cells derived from tumors and tumor-adjacent lung tissues**

(a) UMAP plots showing the expression of cytotoxic T, regulatory T, naïve and exhausted T markers respectively in all the T lymphocytes.

(b) Violin plots showing that LUAD-derived T cells (indicated as “T”) express less effector T marker genes but more regulatory and exhausted genes compared to tumor-adjacent lung-derived T cells (indicated as “N”). GZMA (N v.s. T) LogFoldChange= 0.332487442, adjusted p value= 5.17E^-168^; GZMB (N v.s. T) LogFoldChange=0.142534272, adjusted p value=3.26E^-42^; FOXP3 (T v.s. N) LogFoldChange=0.082017301, adjusted p value=7.92E^-13^; CTLA4 (T v.s. N) LogFoldChange=0.101004531, adjusted p value=4.63E^-13^; TIGIT (T v.s. N) LogFoldChange= 0.258224344, adjusted p value= 2.75E^-143^, and LAYN (T v.s. N) LogFoldChange=0.071034001, adjusted p value=1.90E^-12^.


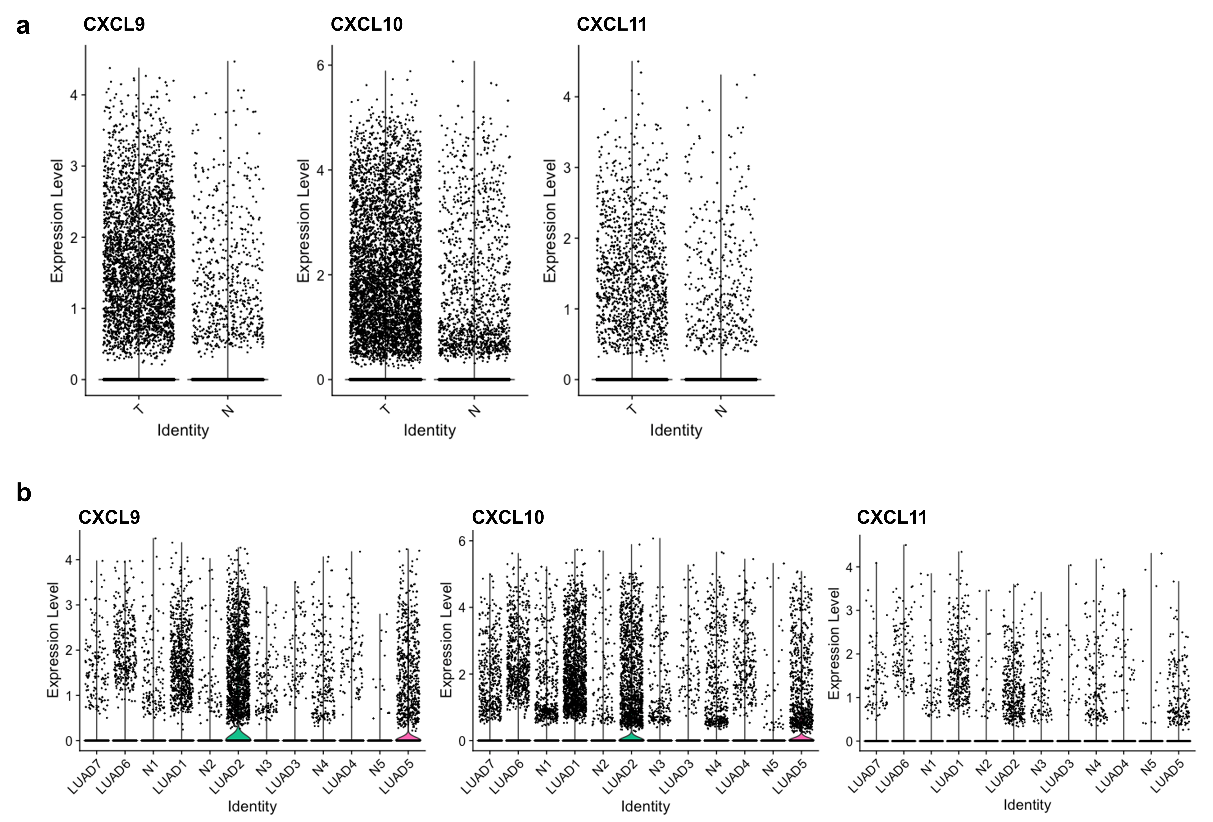


**Figure S5 *CXCL9*, *CXCL10* and *CXCL11* were upregulated in TAMs**

Violin plots showing that T lymphocyte recruiting chemokines CXCL9, CXCL10 and CXCL11 were upregulated in tumor associated macrophages compared to macrophages derived from tumor-adjacent lung tissues (LogFoldChange= 0.4858127, 0.7869661, and 0.3042152 respectively, all adjusted p value< 2.225074e^-308^), suggesting that the macrophages in the TME could result in T cell chemotaxis.


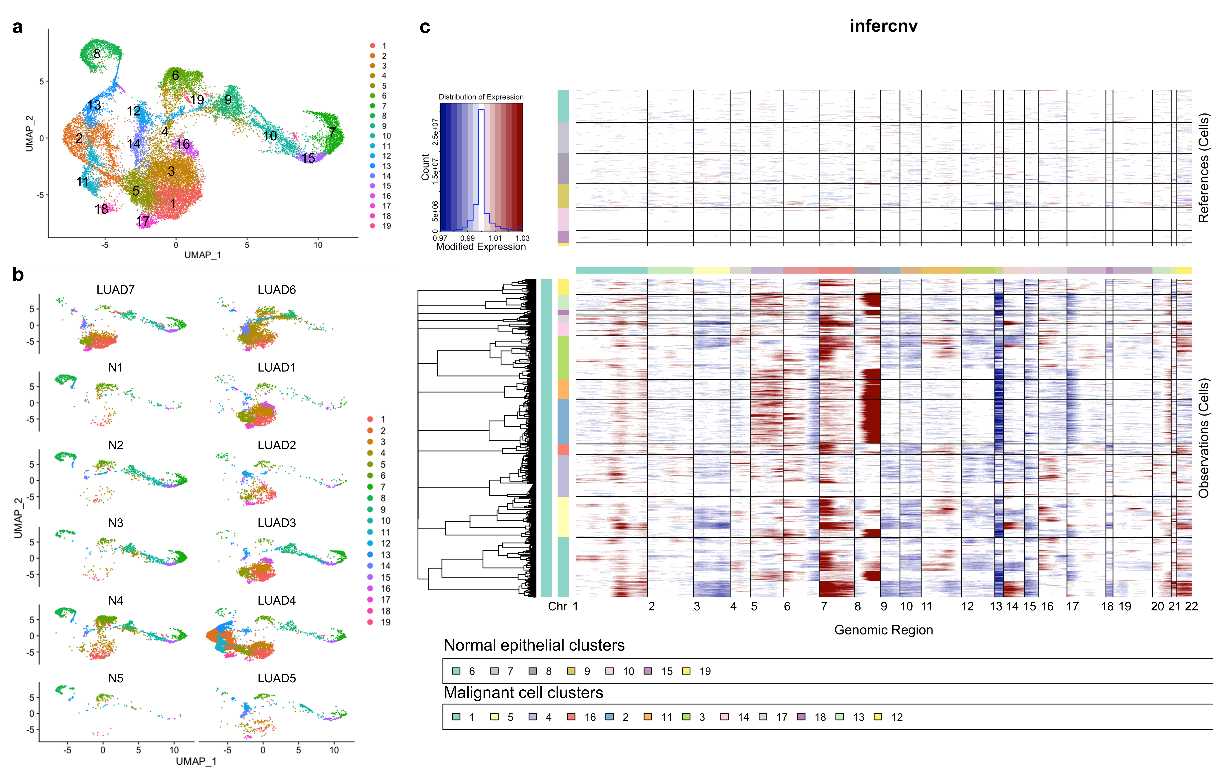


**Figure S6 Identification of malignant cells within tumor samples by inferring copy number variations (CNVs) derived from scRNA-seq data**

(a) UMAP plot showing all the normal lung-derived epithelial cells and tumor derived malignant cells grouped and colored by cell clusters.

(b) UMAP plots showing epithelial cells and malignant cells in LUAD1-5 and their matched normal lung tissues. Normal lung epithelial cells could be found in tumor samples, while some malignant cells could be found in tumor adjacent lung tissues.

(c) Heatplot of malignant cells with aberrant CNVs across each chromosome. The top panel is the normal epithelial clusters referred to as background. The bottom panel is the malignant cells with large scale of copy number gains (red) and copy number losses (blue) across their chromosomes.


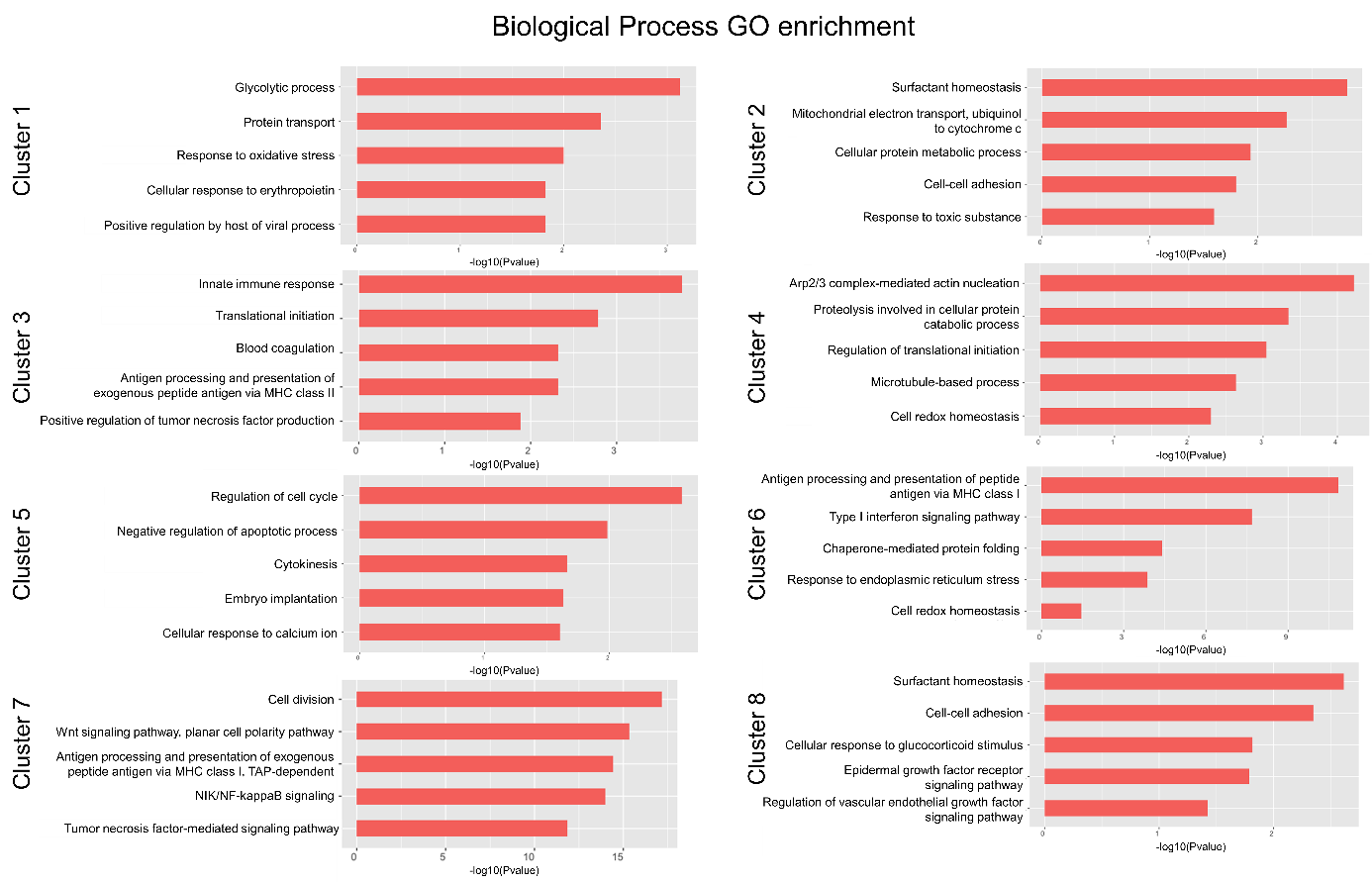


**Figure S7 Representative enriched GO BP terms in DEGs of each tumor subcluster**

Barplots of the enriched Gene Ontology biological process terms in upregulated genes (adjust P value<0.01, log2FC>0.25) of each tumor subcluster.

**
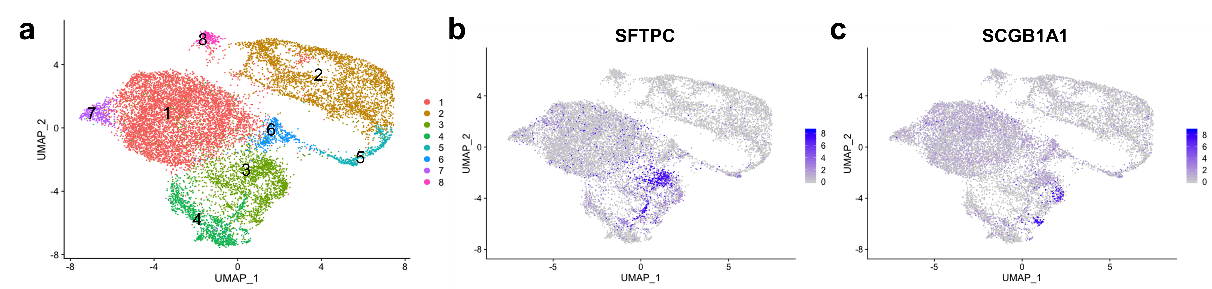
**

**Figure S8 Cluster 3 were selected as root state of the pseudotime trajectory for the expression of normal lung epithelial cell markers**

(a) UMAP plot showing different tumor clusters labeled by different colors.

(b) (c) UMAP plots showing the expression of SFTPC and SCGB1A1 in the tumor cell clusters respectively.

Cells in cluster 3 were chosen to be the root state of the pseudotime trajectory because they still express the markers of the normal lung epithelial cells, i.e., SFTPC and SCGB1A1.

**
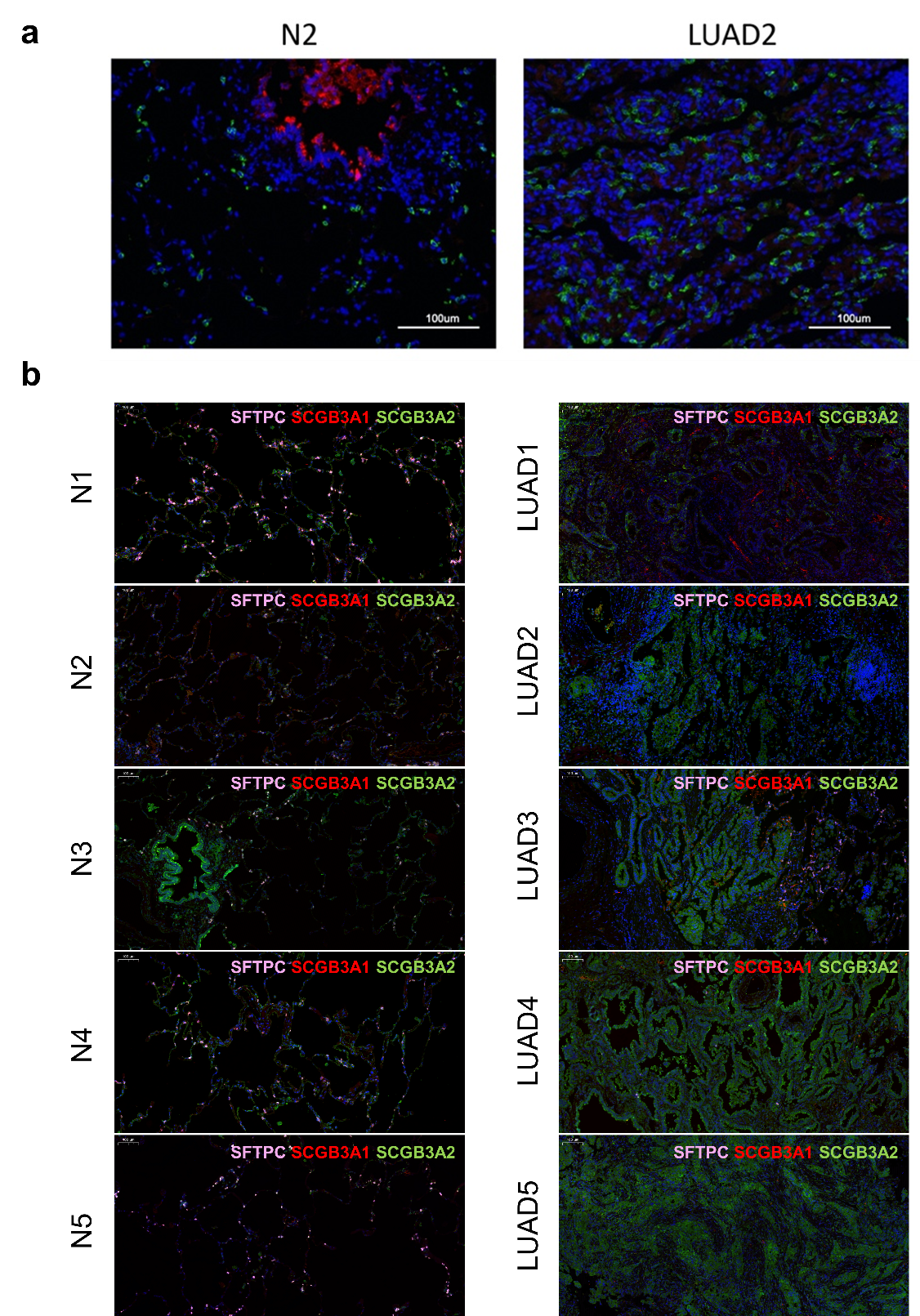
**

**Figure S9 Tumor cells highly express *SCGB3A2***

(a) Representative immunostaining of AT2 cell marker SFTPC and Clara cell marker SCGB1A1 in normal lung (N2) and LUAD (LUAD2) tissues. SFTPC and SCGB1A1 were expressed distinctly in AT2 and Clara cells in normal lung, but their expression colocalized in LUAD tumor cells.

(b) The immunostaining showing that AT2 cell marker SFTPC (pink) was rarely expressed in the tumor tissues. SCGB3A1 (red) was slightly downregulated in the tumor cells compared to the normal alveolar cells, whereas SCGB3A2 (green) was highly expressed in the tumor cells.

**Supplementary Table** **S1, S2, S3, S4 and S7**

**(Table S5 and S6 are in Excel format in separate files.)**

| **Table S1 M1 and M2 signature genes used to generate scatterplot in Figure 2G** | | |
| --- | --- | --- |
| M1 signature genes | IL1B, IL1A, TNF, IL6, CXCL9, CXCL10, IL12A, IL12B, IL23A, FCGR1A, FCGR1B, FCGR1C, CCR7, IL8, CCL5, HLA-DRA, IRF5, IRF1 |  |
| M2 signature genes | IL10, CD163, MARCO, MRC1, MSR1, ARG1, STAB1, TGM2, MMP7, MMP9, MMP19, TGFB1, TGFB2, TGFB3, VEGFA, FN1, CCL4, CCL22, CCL17, CCL18, IL4R, IL7R, IRF4 |  |

| **Table S2 qPCR Primers** | |
| --- | --- |
| Primer | Sequence |
| ELF3-F | CAGATGTCATTGGAGGGTACAG |
| ELF3-R | TCGTGAGAAGTCAATGGCG |
| NFKB1-F | GAACCACACCCCTGCATATAG |
| NFKB1-R | GCATTTTCCCAAGAGTCATCC |
| GADD45B-F | GGGAAGGTTTTGGGCTCTC |
| GADD45B-R | GGTCACCGTCTGCATCTTC |
| BCL2L1-F | AGCCTTGGATCCAGGAGAA |
| BCL2L1-R | AGCGGTTGAAGCGTTCCT |
| CCND1-F | CCTCGGTGTCCTACTTCAAATG |
| CCND1-R | GCGGTCCAGGTAGTTCATG |
| PTGS2-F | ACAGGCTTCCATTGACCAG |
| PTGS2-R | TCACCATAGAGTGCTTCCAAC |
| ICAM1-F | TGACCGTGAATGTGCTCTC |
| ICAM1-R | CTGTATTTCTTGATCTTCCGCTG |
| VEGFA-F | AGTCCAACATCACCATGCAG |
| VEGFA-R | TTCCCTTTCCTCGAACTGATTT |
| MMP9-F | CGAACTTTGACAGCGACAAG |
| MMP9-R | CACTGAGGAATGATCTAAGCCC |

| **Table S3 Statistics of scRNA-seq** | | | | | |
| --- | --- | --- | --- | --- | --- |
| **Sample** | **Number of cells (unfiltered)** | **Number of cells (filtered)** | **Number of reads** | **Mean reads per cell** | **Median genes per cell** |
| LUAD1 | 15,170 | 13,239 | 316,998,109 | 20,896 | 1,187 |
| LUAD2 | 12,011 | 10,562 | 273,486,403 | 22,769 | 1,178 |
| LUAD3 | 11,948 | 6,243 | 332,472,619 | 27,826 | 1,498 |
| LUAD4 | 17,730 | 10,620 | 224,136,308 | 12,641 | 870 |
| LUAD5 | 10,804 | 10,004 | 271,224,208 | 25,104 | 1,040 |
| LUAD6 | 20,497 | 14,822 | 735,006,623 | 35,859 | 489 |
| LUAD7 | 13,914 | 9,008 | 855,553,367 | 61,488 | 1,314 |
| N1 | 13,761 | 12,836 | 283,504,723 | 20,602 | 1,539 |
| N2 | 11,237 | 9,548 | 264,131,622 | 23,505 | 1,016 |
| N3 | 12,048 | 11,089 | 302,695,222 | 25,124 | 1,566 |
| N4 | 11,680 | 10,471 | 234,158,085 | 20,047 | 1,142 |
| N5 | 7,506 | 7,232 | 245,328,447 | 32,684 | 927 |

| **Table S4 Canonical markers used to annotate each cell type cluster** | | |
| --- | --- | --- |
| Cell type | Marker genes | References |
| Monocytes/ macrophages | FCGR3A, ITGAX, ITGAM, CD14, CSF1R, FCGR1A, CCR2, CX3CR1, CD68, CD163, MARCO, MSR1, MRC1, APOE, MCEMP1 | (1-7) |
| T cells | CD3D, CD3E, CD3G, CD2, CD27, CD28, TRAC, TRBC1, TRBC2, CD69 | (5-8) |
| Dendritic cells | CD1A, CD1C, CD207, HLA-DQ, CD103, CD123, CCL17, CCL22 | (6,7,9) |
| B cells | CD79A, CD79B, IGHG1, IGHA1, IGHM, CD19 | (6,7) |
| Mast cells | TPSAB1, TPSB2, CPA3, MS4A2, CLU | (6,7) |
| Ciliated cells | FOXJ1, CAPS, | (10-12) |
| Clara cells | SCGB1A1, SCGB3A1, SCGB3A2 | (10,13) |
| AT1 cells | PDPN, AGER, CAV1 | (10,12) |
| AT2 cells | SFTPC, SFTPB, SFTPD, SFTPA1 | (10) |
| CAFs | COL1A1, COL1A2, THY1, FAP | (7) |
| Endothelial cells | CLDN5, PECAM1 | (12) |

| **Table S7 Additional patient information in studying ELF3 function** | | | | | | | |
| --- | --- | --- | --- | --- | --- | --- | --- |
| No. | Sex | Age | TNM | Stage | Pathology | Driver mutation | Affected lobe |
| T1 | M | 63 | T1N1M1 | IIB | Adenocarcinoma | ND | Right upper |
| T2 | M | 46 | T1N0M0 | IA | Adenocarcinoma | ND | Left upper |
| T3 | F | 67 | T2aN2M0 | IIIA | Adenocarcinoma | EGFR 19del | Right upper |
| T4 | F | 50 | T1bN0M0 | IA | Adenocarcinoma | EGFR 19del | Right upper |
| T5 | F | 56 | T1bN2M0 | IIIA | Adenocarcinoma | EGFR 19del | Right upper |
| T6 | F | 50 | T1N0M0 | IA | Adenocarcinoma | NA | Left upper |
| T7 | F | 60 | T2bN1M0 | IIB | Adenocarcinoma | EGFR 19del | Left lower |
| T8 | F | 51 | T2aN2M0 | IIIA | Adenocarcinoma | ND | Right upper |
| T9 | F | 48 | T1bN1M0 | IIA | Adenocarcinoma | EGFR 19del | Left upper |
| T10 | F | 46 | T1bN0M0 | IA | Adenocarcinoma | ND | Right mid-upper |
| T11 | M | 63 | T2bN2M0 | IIIA | Adenocarcinoma | EGFR 19del | Left upper |
| T12 | M | 59 | T2aN2M0 | IIIA | Adenocarcinoma | EGFR 19del | Right upper |

ND: No mutation detected in BRAF, ROS1, EGFR, KRAS, ELM4-ALK loci

NA: Not Applicable (not screened for mutations).

**Supplementary References**

1. Noy R, Pollard JW. Tumor-associated macrophages: from mechanisms to therapy. Immunity **2014**;41:49-61

2. Qian BZ, Pollard JW. Macrophage diversity enhances tumor progression and metastasis. Cell **2010**;141:39-51

3. Gordon S, Taylor PR. Monocyte and macrophage heterogeneity. Nature reviews Immunology **2005**;5:953-64

4. Chittezhath M, Dhillon MK, Lim JY, Laoui D, Shalova IN, Teo YL*, et al.* Molecular profiling reveals a tumor-promoting phenotype of monocytes and macrophages in human cancer progression. Immunity **2014**;41:815-29

5. Lavin Y, Kobayashi S, Leader A, Amir ED, Elefant N, Bigenwald C*, et al.* Innate Immune Landscape in Early Lung Adenocarcinoma by Paired Single-Cell Analyses. Cell **2017**;169:750-65.e17

6. Bindea G, Mlecnik B, Tosolini M, Kirilovsky A, Waldner M, Obenauf AC*, et al.* Spatiotemporal dynamics of intratumoral immune cells reveal the immune landscape in human cancer. Immunity **2013**;39:782-95

7. Schelker M, Feau S, Du J, Ranu N, Klipp E, MacBeath G*, et al.* Estimation of immune cell content in tumour tissue using single-cell RNA-seq data. Nature communications **2017**;8:2032

8. Zheng C, Zheng L, Yoo JK, Guo H, Zhang Y, Guo X*, et al.* Landscape of Infiltrating T Cells in Liver Cancer Revealed by Single-Cell Sequencing. Cell **2017**;169:1342-56.e16

9. Worbs T, Hammerschmidt SI, Forster R. Dendritic cell migration in health and disease. Nature reviews Immunology **2017**;17:30-48

10. Treutlein B, Brownfield DG, Wu AR, Neff NF, Mantalas GL, Espinoza FH*, et al.* Reconstructing lineage hierarchies of the distal lung epithelium using single-cell RNA-seq. Nature **2014**;509:371-5

11. Choksi SP, Lauter G, Swoboda P, Roy S. Switching on cilia: transcriptional networks regulating ciliogenesis. Development (Cambridge, England) **2014**;141:1427-41

12. Lambrechts D, Wauters E, Boeckx B, Aibar S, Nittner D, Burton O*, et al.* Phenotype molding of stromal cells in the lung tumor microenvironment. Nature medicine **2018**;24:1277-89

13. Reynolds SD, Reynolds PR, Pryhuber GS, Finder JD, Stripp BR. Secretoglobins SCGB3A1 and SCGB3A2 define secretory cell subsets in mouse and human airways. American journal of respiratory and critical care medicine **2002**;166:1498-509
